# Editorial: Kidney Transplantation and Innate Immunity

**DOI:** 10.3389/fimmu.2020.603982

**Published:** 2020-10-14

**Authors:** Paola Pontrelli, Giuseppe Grandaliano, Cees Van Kooten

**Affiliations:** ^1^ Department of Emergency and Organ Transplantation, University of Bari “A. Moro”, Bari, Italy; ^2^ Department of Translational Medicine and Surgery, Università Cattolica del Sacro Cuore, Rome, Italy; ^3^ Nephrology Unit, Department of Medicine and Surgery, Fondazione Policlinico Universitario “A. Gemelli” IRCCS, Rome, Italy; ^4^ Department of Nephrology, Leiden University Medical Center, Leiden, Netherlands

**Keywords:** kidney transplantation, innate immunity, immune cells, complement system, coagulation cascade

## Introduction

Kidney transplantation is the best treatment for End Stage Renal Disease (ESRD). Although the advances in immunosuppressive drugs and protocols have markedly reduced the incidence and relevance of acute rejection, the outcome of kidney grafts is still significantly influenced by the development of chronic cellular or humoral rejection. In this setting adaptive alloimmune response has always been considered as the main, if not the only, player and the role of innate immunity has been disregarded for a long period of time. However, in the last two decades a growing body of evidence suggested that innate immune responses significantly contribute to priming of the rejection machinery and control the activation of alloantigen-specific adaptive immunity.

The principal constituents of innate immunity are represented by cellular components such as phagocytic cells (neutrophils, macrophages), dendritic cells, natural killer (NK) and other innate lymphoid cells, and blood proteins, including members of the complement system and other mediators of inflammation.

Several factors can induce the activation of innate immune responses in clinical kidney transplantation. In Donation after Brain Death (DBD), brain death itself can promote the systemic production and release of pro-inflammatory cytokines such as monocyte chemotactic peptide-1 and interleukin-6, leading to the activation of innate immune pathways such as monocyte recruitment and activation in several organs including the kidneys. Moreover, warm and cold ischemia during kidney retrieval and preservation followed by reperfusion at the time of transplantation is also known to induce, through several molecular mechanisms including oxidative stress and resident cells apoptosis, the activation of innate immune responses. Interestingly, the communication between innate and adaptive immune response in kidney graft rejection is bi-directional since there is now clear evidence that adaptive immune response activation may lead to tissue damage through cellular and molecular components of innate immunity.

The 17 articles of this Research Topic summarize recent achievements and provide timely reviews in the field of the different components of innate immunity and kidney transplantation.

## Innate Immune Cells in Kidney Transplantation

Different cell types involved in the innate immune response can play an important role in kidney transplantation, and are influenced by the suppression of the recipient’s immune. Recognition of allogeneic non-self induces the activation of recipients T lymphocytes, that can inflict direct cytotoxicity on graft cells or can influence other cells of the immune system such as B lymphocytes or macrophages. In this scenario also dendritic cells might play an important role as antigen presenting cells, and their activation can influence innate immune responses in different ways, for example, through activation of natural killer cells. In the present issue, six papers described the characteristic and behavior of different innate immune cells in the setting of kidney transplantation and graft rejection. This section highlights that basic knowledge on innate immune cells in kidney transplantation is continuously emerging with the description of novel functions relying on recently identified cells subsets or on cross-talk with distinct other cell types.

Sablik et al. performed a detailed identification of inflammatory cells in renal allograft biopsies from patients with chronic-active antibody-mediated rejection through multiplex immunofluorescent staining. The glomerular compartment was predominantly characterized by CD8+ cytotoxic T cells (granzyme B+ and CD57+) and M2 macrophages (CD68+ and CD163+). T cells (either CD4+ or CD8+ T cells) and macrophages were also present in the tubular interstitial compartment but relatively few CD8+ T cells expressed granzyme and/or CD57. In the tubular compartment there were also CD3+FoxP3+ cells, and their increased number was significantly associated with a poor renal allograft survival.

Dai et al. provided an up-date on the role of dendritic cells in ischemia injury. Their review includes a description of the dendritic cells “interactome” following renal ischemia reperfusion injury, finally leading to renal tubular epithelial cell apoptosis, glomerular endothelial damage, and fibrin deposition. During post-ischemia reperfusion injury, intra-renal dendritic cells can activate natural killer T cells and amplify the innate immune response. Ischemia reperfusion injury is responsible of increased inflammation in kidney transplantation thus mediating the recruitment of different cell-types, included B cells. In their research, Kreimann et al. observed an increase in the number of cells expressing the CXCL13 receptor CXCR5, the majority of which were B-cells, through a single-cell sequencing analysis. In a mouse model of kidney transplantation, they confirmed that increased levels of systemic serum CXCL13 correlated with length of cold ischemia time.

Ischemia/reperfusion injury induces an inflammatory reaction that is mediated by Pattern Recognition Receptors expressed on both infiltrating immune cells and tubular epithelial cells. In their review Tammaro et al. described how engagement of these innate immune receptors can influence behavior of tubular cells in terms of mitochondrial dysfunction, senescence, cell death and production of pro-fibrotic cytokines. Cross-talk between innate immunity and tubular cell metabolism represents a novel approach in the evaluation of cell fate in ischemia-reperfusion injury.

Among the different cell types involved in the innate immune response, also natural killer cells subsets play an important role in kidney graft damage. The knowledge on natural killer cells has greatly evolved recently and Pontrelli et al. provided an update on their role in kidney transplantation. Their review includes the description of the different natural killer subsets involved in antibody and T-Cell mediated rejection, but also how natural killer cells can contribute to transplant tolerance and the influence of immunosuppression on natural killer cell phenotype. The specific phenotype of infiltrating natural killer cells in human kidney allograft rejection has been confirmed by Kildey et al. through an innovative multi-color flow cytometry-based approach, confirming that natural killer cell subsets are differentially recruited and activated during distinct types of rejection.

## Cross-Talk Between Complement, Coagulation, and Innate Immune Cells in Kidney Transplantation

Innate immune responses in kidney transplantation are also characterized by the activation of complement system, as well as of the coagulation cascade. These key components of innate immunity are tightly connected to each other. The complement system is a crucial mediator of the innate immune response, thus influencing other endogenous systems. The complement cascade can be activated by three major complement activation pathways (classical, alternative, and lectin pathway) that converge into a common sequence leading to the formation of C3- and C5-convertases and generation of the anaphylatoxins C3a and C5a and of the C5b-9 membrane attack complex. The complement system is highly regulated to prevent over-activation, since this may lead to systemic inflammation, dysregulation of coagulation/fibrinolysis, and tissue-damage thus contributing to allograft injury.

In their review, Grafals et al. analyzed how the complement system can influence inflammatory injury of the graft and the response of B and T cells to donor antigens. They also explored the role of complement inhibitory drugs in preventing immune responses against allografts, suggesting their possible use as adjuncts to the currently available anti-rejection drugs in kidney transplantation.

Complement activation in kidney transplantation can be induced by donor-brain death and is associated with a worst renal allograft outcome. In their paper, Jager et al. investigated the role of the alternative pathway in a model of brain-death induced in Fisher rats. Pre-treatment of rats with anti-factor B exhibited unique complement-regulatory and anti-inflammatory properties and thus extends the emerging field of complement therapeutics. Activation of complement may contribute to the progression of renal failure through tubular C5b-9 formation. Lammerts et al. investigated the alternative pathway complement factor properdin and the terminal sC5b-9 complex in the urine of a previously described cohort of 707 renal transplant recipients. They observed that after kidney transplantation, independent of proteinuria, the urinary presence of properdin and the terminal sC5b-9 complex showed significant impact on the rate of graft failure and graft survival, suggesting them as useful biomarkers of immunological injury and kidney allograft deterioration.

Wang et al. highlighted the role of increased labile heme levels in the kidney, due to prolonged warm ischemia, in the up-regulation of renal inflammation and activation of the complement system. In their paper they used a mouse model of ischemia-reperfusion-injury to demonstrate that prolonged ischemia-reperfusion injury not only increased labile heme concentrations in renal tissue, but also up-regulated C5a receptor, as well as several pro-inflammatory and pro-fibrotic cytokines and induced neutrophil infiltration. Interestingly, heme removal by human serum albumin reduced the expression of pro-inflammatory cytokines, C3a receptor and improved tubular function after ischemia-reperfusion injury.

Beside the pivotal role in innate immunological response, complement activation is also involved in the aging process. The review from Franzin et al. highlights the link existing between complement activation and premature renal senescence in the context of the transition from acute kidney injury to chronic kidney disease, with a special focus on ischemia/reperfusion Injury and antibody-mediated rejection. Strategies to target complement in kidney transplantation, to prevent the development of acute kidney injury and its progression to chronic disfunction, were also discussed both at the experimental and clinical level. Finally, authors provide emerging insights on molecular mechanisms involved in complement-induced renal “inflammaging,” including Klotho signaling, Wnt/β catenin pathway, epigenetic changes, and cell cycle arrest.

Systemic activation of the complement cascade as well as of the coagulation system, can be modulated by extracellular vesicles that are known immune-modulators and might play a critical role in kidney transplantation. In their review Quaglia et al. described the involvement of extracellular vesicles in the modulation of innate/adaptive immune systems and their role as shuttle of specific mediators involved in graft tissue injury. Investigation of extracellular vesicles in urinary and serum samples is a very interesting approach for the identification of potential biomarkers of graft damage. Extracellular vesicles might also represent promising therapeutic tools in kidney transplantation, functioning as vehicle of drugs or miRNAs to antagonize specific mediators of inflammation or graft damage.

Stallone et al. highlighted the role of the coagulation cascade and fibrinolytic system in the ischemia/reperfusion injury and in the pathogenesis of tissue damage in acute and chronic rejection. Following ischemia-reperfusion injury, the coagulation cascade is strongly activated, mainly induced by the vascular expression of tissue factor. Fibrin deposition in the kidney graft may also represent a challenging cause of graft dysfunction thus leading to graft rejection. Several interactions between coagulation, fibrinolysis and complement have been proposed in a variety of clinical conditions, including kidney transplantation, opening the way for inhibition of coagulation to modulate innate immunity and to prevent progressive graft damage.

## Innate Immune Cells and Therapy

Innate immune cells such as dendritic cells, monocytes, macrophages, neutrophils and natural killer cells play an important role in most immunological events following kidney transplantation and their behavior can be significantly influenced by immunosuppressive therapies. The definition of the therapeutic range of specific drugs is the final goal for each clinician in order to obtain better outcomes of the graft with reduced adverse events such as graft rejection or occurrence of infections.

Song et al. investigated how the duration of time being within the therapeutic range of tacrolimus-based immunesuppressive regimen might influence the long-term clinical outcomes in living kidney transplantation. Since there is a considerable association between the mantainance within the tacrolimus therapeutic range in the first year and improved long-term outcomes in living kidney transplants, this is a very interesting approach for the future monitoring of tacrolimus exposure.

Differences in therapy regimen can influence cell phenotype and biological effects. Pilon et al. performed a monocentric prospective cohort study of kidney allograft recipients with *de novo* DSA, to evaluate transcriptomic and phenotypic changes in T and B lymphocytes as well as serum cytokines after treatment with high dose intravenous immunoglobulin. High dose intravenous immunoglobulin induced limited modifications in B and T cell phenotype, however results need to be confirmed in a larger population in order to evaluate the clinical use of high dose intravenous immunoglobulin after kidney transplantation.

In their review Zaza et al. discussed, in the setting of kidney transplantation, the effects of currently used immunosuppressive agents on innate immune cells, their direct effects and the effects on induced adaptive immune response. Their overview highlights the possibility that novel drug candidates targeting innate immune cells could be considered in order to prolong allograft function and minimize immunosuppression

One of the most common infections in kidney transplanted patients due to immunosuppression regimen, is represented by Cytomegalovirus. Notably, in the paper by Rahmel et al. authors demonstrated an association between the genetic background of the Aquaporin 5 gene with infection risk, in a cohort of kidney or combined pancreas–kidney transplanted patients from a single center. The presence of a single nucleotide polymorphism in the promoter region of the Aquaporin 5 gene represents an independent risk-factor of post-transplant infections and could open the way to novel strategies for post-transplantation Cytomegalovirus prophylaxis.

## Conclusion

We hope that this Research Topic will highlight the importance of innate immunity contributing to the mechanisms involved in graft deterioration and rejection and will open the way to novel therapeutic approaches in the management of kidney transplant recipients.

## Author Contributions

All authors contributed to the article and approved the submitted version.

## Conflict of Interest

The authors declare that the research was conducted in the absence of any commercial or financial relationships that could be construed as a potential conflict of interest.

